# Phytochemicals of Cinnamomi Cortex: Cinnamic Acid, but not Cinnamaldehyde, Attenuates Oxaliplatin-Induced Cold and Mechanical Hypersensitivity in Rats

**DOI:** 10.3390/nu11020432

**Published:** 2019-02-19

**Authors:** Hyeon Kyeong Chae, Woojin Kim, Sun Kwang Kim

**Affiliations:** 1 Department of Science in Korean Medicine, Graduate School, Kyung Hee University, Seoul 02447, Korea; hyeonkyeong.chae@gmail.com; 2 Department of Physiology, College of Korean Medicine, Kyung Hee University, Seoul 02447, Korea

**Keywords:** Cinnamomi Cortex, phytochemical, cinnamic acid, cinnamaldehyde, oxaliplatin, neuropathic pain, allodynia, spinal cord, wide dynamic range neuron

## Abstract

A chemotherapy drug, oxaliplatin, induces cold and mechanical hypersensitivity, but effective treatments for this neuropathic pain without side effects are still lacking. We previously showed that Cinnamomi Cortex suppresses oxaliplatin-induced pain behaviors in rats. However, it remains unknown which phytochemical of Cinnamomi Cortex plays a key role in that analgesic action. Thus, here we investigated whether and how cinnamic acid or cinnamaldehyde, major components of Cinnamomi Cortex, alleviates cold and mechanical allodynia induced by a single oxaliplatin injection (6 mg/kg, i.p.) in rats. Using an acetone test and the von Frey test for measuring cold and mechanical allodynia, respectively, we found that administration of cinnamic acid, but not cinnamaldehyde, at doses of 10, 20 and 40 mg/kg (i.p.) significantly attenuates the allodynic behaviors in oxaliplatin-injected rats with the strongest effect being observed at 20 mg/kg. Our in vivo extracellular recordings also showed that cinnamic acid (20 mg/kg, i.p.) inhibits the increased activities of spinal wide dynamic range neurons in response to cutaneous mechanical and cold stimuli following the oxaliplatin injection. These results indicate that cinnamic acid has an effective analgesic action against oxaliplatin-induced neuropathic pain through inhibiting spinal pain transmission, suggesting its crucial role in mediating the effect of Cinnamomi Cortex.

## 1. Introduction

Oxaliplatin is a widely used third-generation platinum chemotherapeutic agent usually prescribed with fluorouracil and leucovorin against metastatic colorectal [1,2], breast, ovarian and lung cancer [3]. Furthermore, oxaliplatin has no cross-resistance and has a higher efficacy and a lower nephrotoxicity than cisplatin, which is a first generation platinum chemotherapeutic agent [4]. However, the acute neuropathic pain induced by even a single treatment of oxaliplatin affect more than 90% of treated patients [5]. This oxaliplatin-induced neuropathic pain is characterized by peripheral paresthesia and dysesthesia of hands and feet [5,6]. Especially, cold and mechanical hypersensitivities are emphasized [7,8].

This neuropathic pain can interrupt the treatment schedule, and put an end to treatments by deteriorating the patient’s quality of life [5,9,10]. Today, drugs used as first-line therapy for this pain are selective serotonin, norepinephrine reuptake inhibitors (duloxetine, venlafaxine) and anticonvulsants (gabapentin, pregabalin) [11]. However, these drugs also have untoward side effects such as headache, dizziness and somnolence, which limit their use [12,13]. Thus, it is important to put efforts to find out new alternative medicine that could attenuate oxaliplatin-induced neuropathic pain without causing side effects.

In our previous study, oral administration of water extract of Cinnamomi Cortex (C. Cortex; *Cinnamomum cassia* P., family Lauraceae) significantly attenuated cold allodynia induced by single oxaliplatin injection [14]. By using ultra-high performance liquid chromatography, we confirmed that C. Cortex is mainly composed of coumarin, cinnamic acid and cinnamaldehyde. To further understand which phytochemical plays a major role in the analgesic effect of C. Cortex, we first tested the effect of coumarin among the three main phytochemicals. As a result, we found out that five consecutive oral administrations of coumarin (10 mg/kg) significantly attenuated the cold allodynia induced by oxaliplatin [14]. However, coumarin did not attenuate the mechanical allodynia whereas C. Cortex significantly decreased both the cold and mechanical allodynia (unpublished data). Moreover, coumarin has been reported to cause hepatotoxicity in rats [15].

Thus, in this study, we first determined whether cinnamic acid (CA) or cinnamaldehayde (CD) plays a key role in mediating the suppressive effect of C. Cortex on oxaliplatin-induced cold and mechanical allodynia. Secondly, we verified its effect on cutaneous stimuli-evoked neuronal hyperexcitation in the spinal cord of oxaliplatin administered rats, using in vivo extracellular recordings.

## 2. Materials and Methods

### 2.1. Animals

Seven-week-old, male Sprague Dawley (SD) rats (180–210 g, *n* = 95 in total, Young Bio, Gyeonggi, Korea) were housed in a cage with free supply of food and water. The room temperature was maintained at 23 ± 2 °C and kept on a 12-h/12-h light-dark cycle. All procedures described on this study were approved by the Institutional Animal Care and Use Committee of Kyung Hee University (KHUASP[SE]-18-153) and were performed according to the institutional guidelines of the International Association for the Study of Pain [16,17].

### 2.2. Behavioral Tests and Experimental Protocols

To measure cold and mechanical allodynia, we used acetone drop test and von Frey filament (Linton Instrumentation, Norfolk, UK) test (up-down method), respectively [18]. Rats were habituated to handling and to all test procedures for a week before the initiation of the experiments. The experimenters were blinded to oxaliplatin and any other drug injections. Rats were placed in a clear plastic box (20 × 20 × 14 cm) with a wire mesh floor and habituated for 30 min before testing.

For cold allodynia test, acetone (10 μL) was treated to the ventral surface of the right hind paw by pipet connected rubber tube, and the behavioral responses were monitored for 20 s. Acetone was applied five times to the right hind paw, and the total frequencies of licking and flicking were averaged per set.

For mechanical allodynia test, paw withdrawal thresholds were measured in right hind paw using the von Frey filaments. Dixon’s up-down method and Chaplan’s calculation methods were used and withdrawal threshold of 15 g was applied as the cut-off [19,20].

To see the time-dependent effects of different doses of two phytochemicals, CA and CD, behavioral tests were conducted at time point zero (before the administration of chemicals), 30, 60, and 120 min after the injection.

### 2.3. Oxaliplatin Administration

Oxaliplatin (Wako Pure Chemical Industries, Osaka, Japan) was dissolved in a 5% glucose solution at a concentration of 2 mg/mL and injected intraperitoneally (i.p.) at a dose of 6 mg/kg. The control group received the same volume of 5% glucose solution (i.p.) [17].

### 2.4. Cinnamic Acid and Cinnamaldehyde Administration

CA (*trans*-Cinnamic acid, Wako Pure Chemical Industries, Osaka, Japan) and CD (Cinnamaldehyde, Wako Pure Chemical Industries, Osaka, Japan) were dissolved in 10% dimethyl sulfoxide (DMSO, Sigma) (adjusted pH 7 by using 2M HCl and 5M NaOH) and 1% Tween 20 (Sigma) respectively. The final volume of 10% DMSO and 1% tween 20 used in the experiments is 2 µL/g rat. Different doses of CA and CD (10, 20, and 40 mg/kg) were administered (i.p.) in rats with cold and mechanical allodynia.

### 2.5. In Vivo Extracellular Recording

Extracellular recordings were performed on day four after the administration of oxaliplatin, when rats exhibited significant mechanical and cold allodynia. As previously described [21], rats were anesthetized with urethane (Sigma; 1.5 g/kg, i.p.) and the spinal cords of the animals, which were fixed in a stereotaxic frame, were exposed from T13–L2 and irrigated with oxygenated (95% O_2_-5% CO_2_ gas) Krebs solution (in mM: 117 NaCl, 3.6 KCl, 2.5 CaCl_2_, 1.2 MgCl_2_, 1.2 NaH_2_PO_4_, 11 glucose, and 25 NaHCO_3_) at a flow rate of 10–15 mL/min at 38 ± 1 °C. Based on their responses to brush, pressure, pinch, and acetone stimulations, the wide dynamic range (WDR) cells were identified. Extracellular single-unit recordings were made with a low-impedance insulated tungsten microelectrode (impedance of 10 MΩ, FHC, Bowdoin, ME, USA). For mechanical stimuli, brush, press, and pinch stimulations were applied to the lateral and ventral surfaces of the hind paw. Brush stimulus was given by brushing the receptive field five times with a camel brush. Press stimulus was given by pressing the receptive field for 4 s using the blunt tip of the camel brush with a diameter of 0.5 cm and a magnitude of about 20 g. Pinch stimulation was given by pinching the skin using toothed forceps (11022-14, Fine Science Tools, Heidelberg, Germany) for 3 s. For cold stimulation, 10 μL of acetone drop was applied to the receptive fields [22].

### 2.6. Statistics

**Figure 1:**
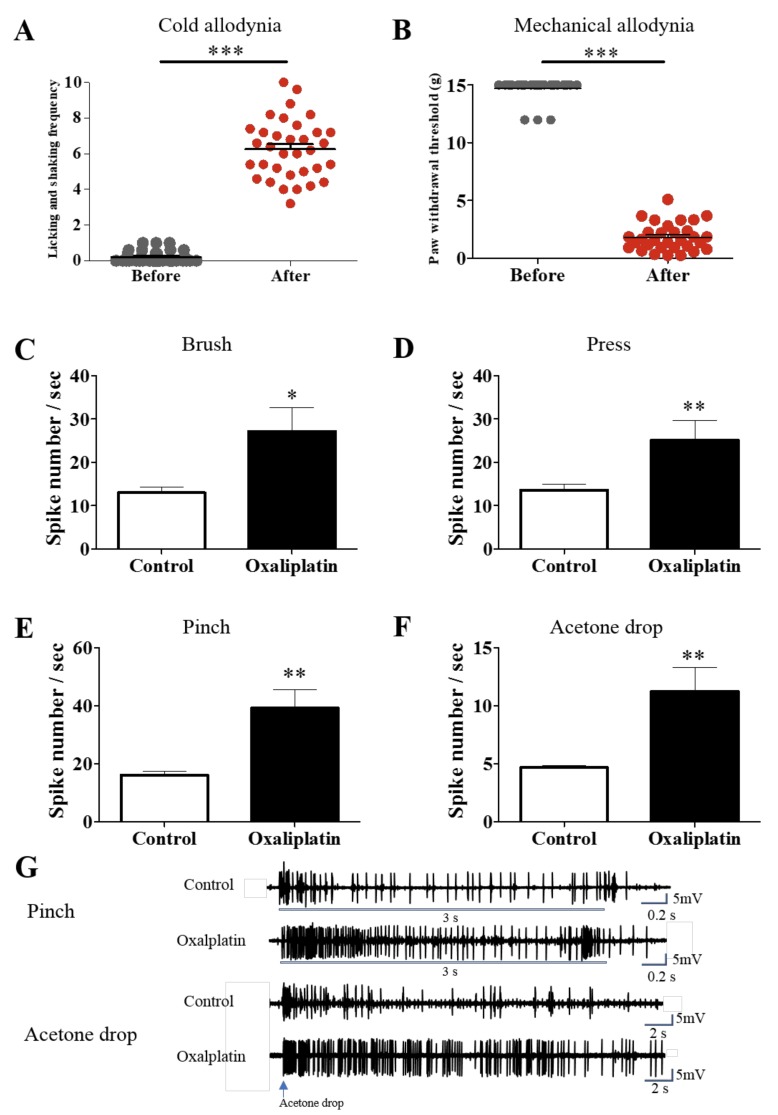
Single oxaliplatin injection induces neuropathic pain behaviors and hyperexcitation of spinal neurons in rats. Four days after single oxaliplatin injection, significant cold; *n* = 34 (**A**) and mechanical; *n* = 31 (**B**) allodynia were induced in rats. Firing frequencies of spinal dorsal horn WDR cells to brush, press, pinch and acetone drop stimuli were measured after 5% glucose (control, *n* = 7) or oxaliplatin (oxaliplatin, *n* = 7) injections (**C–F**). Representative raw trace of WDR neuron firings to pinch and acetone drop stimuli in the control and oxaliplatin groups (**G**). Data is presented as the mean ± S.E.M.; * *p* < 0.05, ** *p* < 0.01, *** *p* < 0.001 vs. Before; by paired *t*-test (**A**,**B**) and * *p* < 0.05, ** *p* < 0.01, *** *p* < 0.001 vs. Control; by unpaired *t*-test **(C–F)**.

**Figure 2:**
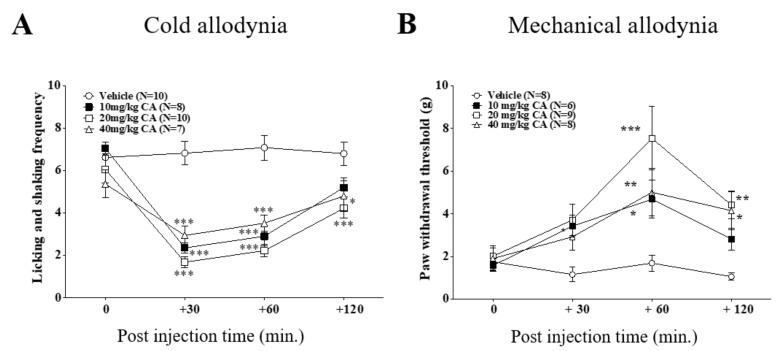
Intraperitoneal administration of cinnamic acid (CA) attenuates oxaliplatin-induced cold and mechanical allodynia. Analgesic effects of three different doses of CA (10, 20, and 40 mg/kg). CA was injected intraperitoneally on day 4, when significant allodynic signs were observable in oxaliplatin treated rats (time point zero). Behavioral tests for both cold (**A**) and mechanical (**B**) allodynia were conducted on 0, 30, 60, and 120 min after CA injection. Vehicle group received 10% DMSO as control. Data is presented as the mean ± S.E.M.; * *p* < 0.05, ** *p* < 0.01, *** *p* < 0.001 vs. 0 min; by Bonferroni post-test after one-way ANOVA (**A**,**B**).

**Figure 3:**
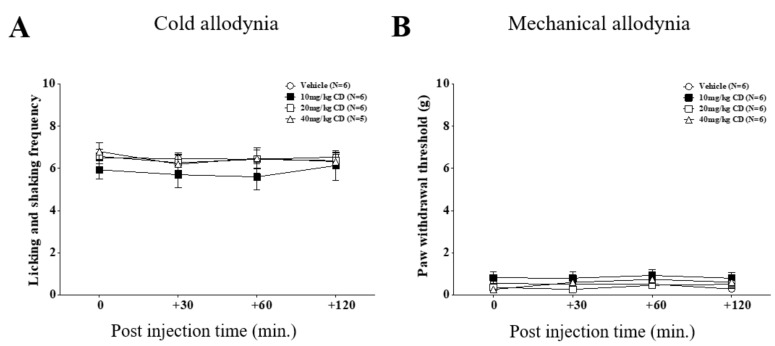
Intraperitoneal administration of cinnamaldehyde (CD) does not affect oxaliplatin-induced allodynia. Analgesic effects of three different doses of CA (10, 20, and 40 mg/kg) are shown. CD was injected intraperitoneally on day 4, when significant allodynic signs were observable in oxaliplatin treated rats. Behavioral tests for both cold (**A**) and mechanical (**B**) allodynia were conducted on time point 0, 30, 60, and 120 min after CD injection. Vehicle group received 1% Tween 20 as control. Data is presented as the mean ± S.E.M.; * *p* < 0.05, ** *p* < 0.01, *** *p* < 0.001 vs. 0 min; by Bonferroni post-test after one-way ANOVA (**A**,**B**).

**Figure 4:**
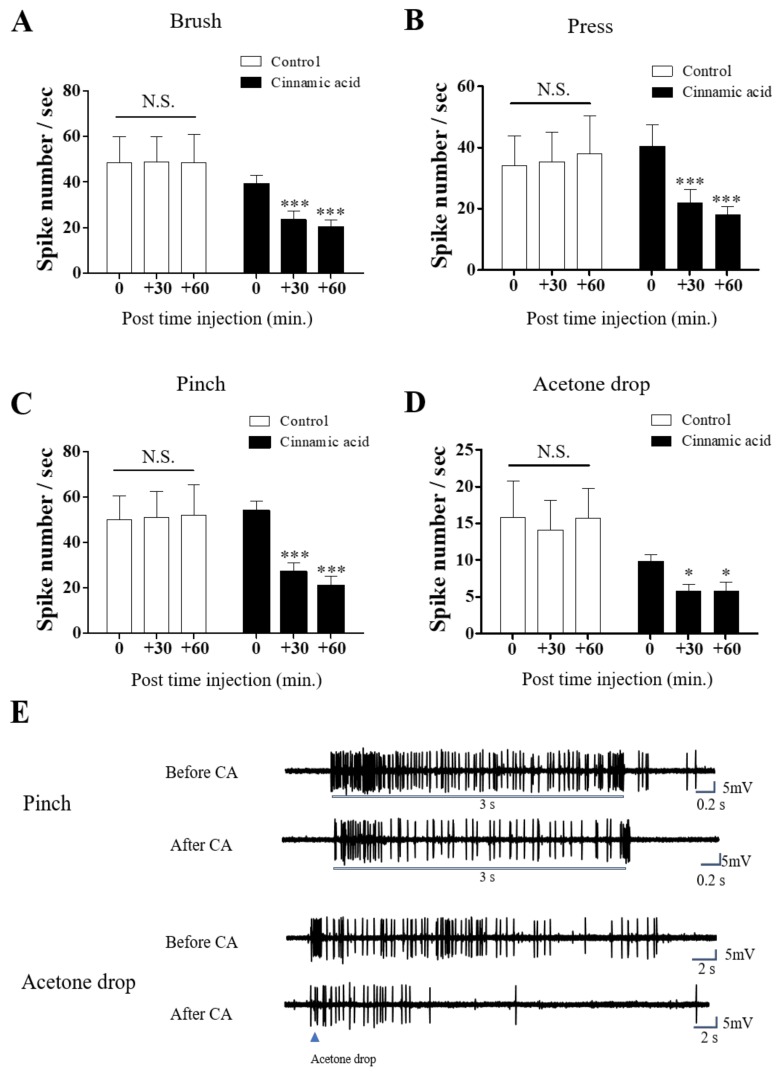
CA inhibits the hyperexcitability of spinal dorsal horn WDR cells to cutaneous stimuli in oxaliplatin treated rats. In vivo extracellular recordings were used to measure the change in firing frequencies of WDR cells to cold and mechanical stimuli after CA administration (20 mg/kg, i.p.) in oxaliplatin injected rats; *n* = 8. Control rats received the same volume of 10% DMSO; *n* = 6. Data is presented as the mean ± S.E.M.; * *p* < 0.05, *** *p* < 0.001 vs. time point zero; by Dunnett’s multiple comparisons test after two-way ANOVA (**A–D**), N.S.; non-significant. (**E**) Representative raw trace of WDR neuron firings in response to pinch or acetone drop stimuli before and after injection of CA.

All the data was represented as the mean ± S.E.M. Data in Figure 1 was analyzed by paired *t*-test (Figure 1A,B) or unpaired *t*-test (Figure 1C–F). Data in Figure 2 and Figure 3 was represented as mean ± S.E.M and was analyzed by Bonferroni post-test after one-way ANOVA to assess statistical differences among the groups. Data in Figure 4A–D was analyzed by using Dunnett’s multiple comparisons test after two-way ANOVA. Confidence of 95% was considered as statistically significant.

## 3. Results

### 3.1. Behavioral and Electrophysiological Correlates of Oxaliplatin-Induced Neuropathic Pain in Rats

A single intraperitoneal injection of oxaliplatin (6 mg/kg) induces neuropathic pain in rats. In our previous studies, significant cold and mechanical allodynia were observed from day three to seven after oxaliplatin injection [22,23]. Figure 1A,B show the results of acetone drop and von-Frey stimuli before and after oxaliplatin injection. Four days after oxaliplatin injection, both the cold and mechanical allodynia were significantly induced (*p* < 0.001). Once the behavioral signs of neuropathic pain were observed, in vivo extracellular recordings were conducted in the rat spinal cord neurons. The number of spike responses of WDR neurons to mechanical (brush, press and pinch) and cold (acetone drop) stimuli were significantly increased after the injection of oxaliplatin (Figure 1C–F), indicating the hyperexcitation of WDR neurons by cutaneous stimuli. Representative raw traces of WDR neuron’s responses to pinch and acetone drop are shown in Figure 1G. These behavioral and electrophysiological results clearly validate the establishment of neuropathic pain four days after the injection of oxaliplatin.

### 3.2. CA Attenuates Oxaliplatin-Induced Cold and Mechanical Allodynia

To observe whether CA could decrease the cold and mechanical allodynia induced by oxaliplatin administration, we conducted behavioral tests with three different doses of CA (10, 20, and 40 mg/kg, i.p.). On cold allodynia, all three doses of CA were shown to be effective; however, the effect of 10 mg/kg of CA was shorter than 20 and 40 mg/kg, as its effect disappeared at 120 min time point. Between 20 and 40 mg/kg, analgesic effect of 20 mg/kg CA was shown to be slightly stronger than 40 mg/kg (control vs. 20 mg/kg, *p* < 0.001 and 40 mg/kg, *p* < 0.05) (Figure 2A). On mechanical allodynia, 10 mg/kg of CA was slightly effective as its effect was significant only at 60 min after injection, however, 20 mg/kg of CA had the strongest anti-allodynic effect among the three doses during all the time points (Figure 2B). These results indicate that intraperitoneal administration of CA potently alleviates oxaliplatin-induced cold and mechanical allodynia in rats, for which the optimal dose is 20 mg/kg.

### 3.3. Cinnamaldehyde Has No Effect on Oxaliplatin-Induced Cold and Mechanical Allodynia

In this study, we also tested the effect of CD, which is another major component of C. Cortex. However, in contrast to CA, three different doses of CD (10, 20, and 40 mg/kg, i.p.) did not show any significant analgesic effect against oxaliplatin-induced cold and mechanical allodynia (Figure 3).

### 3.4. CA Inhibits the Hyperexciation of Spinal WDR Neurons in Oxaliplatin-Injected Rats

To see if CA can reduce the increased activities of WDR neurons after oxaliplatin injection, we conducted in vivo extracellular recordings in the rat spinal cord (see Materials and Methods) on day four, when behavioral and electrophysiological correlates of neuropathic pain were established (Figure 1). CA was given intraperitoneally at a dose of 20 mg/kg, which was optimal for attenuating cold and mechanical allodynia (Figure 2). DMSO (10%, i.p.) was used as a control. Mechanical (brush, press and pinch) and cold (acetone drop) stimuli were given before and after the administration of CA or control. Increased WDR neuronal activities following oxaliplatin injection were significantly decreased after intraperitoneal administration of 20 mg/kg of CA. However, 10% DMSO did not change these increased activities of WDR (Figure 4A–D). Representative raw data exhibiting the decrease of WDR cell’s firings to pinch and acetone drop by CA is shown in Figure 4E. These results suggest that intraperitoneal administration of CA at 20 mg/kg strongly inhibits the hyperexcitation of spinal WDR neurons in rats with oxaliplatin-induced cold and mechanical allodynia.

## 4. Discussion

Oxaliplatin is more widely used compared to the drugs of other generations [24] as it bears no nephrotoxicity and hepatoxicity [25] and its effect against colon carcinoma cell lines resistant to other platinum drugs has been reported [26]. Nevertheless, even a single intraperitoneal injection of oxaliplatin can cause peripheral neuropathic pain [14,17,21], which is a dose-limiting cause [27]. Different categories of analgesic drugs are used to attenuate this neuropathic pain; however, these drugs can also cause side effects, which limit their wide use [12,13]. Thus, efforts to develop a novel drug are continually needed.

In our previous study, we found that C. Cortex can effectively attenuate oxaliplatin-induced cold and mechanical allodynia; however, more experiments had to be conducted to understand which phytochemicals of C. Cortex played a major role in this analgesic effect [14]. Also by using HPLC, it was shown that 0.226% and 0.027% of CA and CD exist in water extract of C. Cortex, respectively. As 400 mg/kg of C. Cortex was administered orally for five consecutive days to decrease oxaliplatin-induced neuropathic pain, approximately 0.9 and 0.1 mg/kg of CA and CD were contained in 400 mg/kg of C. Cortex, respectively. In the present study, we determined the effects of two main components of C. Cortex, CA and CD, on oxaliplatin-induced neuropathic pain behaviors and investigated their electrophysiological mechanism.

As reported from others and our lab [28,29], we showed that single intraperitoneal injection of oxaliplatin (6 mg/kg) can cause significant behavioral signs of cold and mechanical allodynia in rats. Also, by using in vivo extracellular electrophysiology, we showed that spike numbers of spinal WDR neuronal cells in response to cutaneous cold and mechanical stimuli were increased after oxaliplatin injection (Figure 1), validating the establishment of neuropathic pain induced by oxaliplatin in rats. On day four, when the allodynic signs were strongly shown, we administered CA intraperitoneally. Different doses of CA (10, 20 and 40 mg/kg) attenuated cold and mechanical allodynia, but 20 mg/kg produced the strongest analgesic effect (Figure 2). However, on the contrary to CA, all three doses of CD (10, 20 and 40 mg/kg) did not show any analgesic effect against oxaliplatin-induced cold and mechanical allodynia (Figure 3). These results suggest that CA, but not CD, play a key role in the anti-allodynic effect of C. Cortex in oxaliplatin treated rats.

Previously published articles by other research group reported that when CD was injected into naïve rats, it significantly reduced the paw withdrawal latency to cold and mechanical stimuli. They speculated that this result may be due to the agonist action of CD on transient receptor potential ankyrin 1 (TRPA1) [30]. CD was reported to act as a TRPA1 agonist like mustard oil, and activation of TRPA1 was shown to induce cold and mechanical allodynia [31,32]. Thus, this may explain the reason why in our experiment CD did not show any analgesic effect. However, in our study CD did not induce cold and mechanical hypersensitivity as in other papers, and this may be due to the differences in the injected sites (paw vs. intraperitoneal) and pain models (naïve vs. neuropathic pain).

In our last experiment (Figure 4), we showed that CA (20 mg/kg, i.p.) significantly reduces the increased activities of spinal WDR neurons in response to cutaneous stimuli in oxaliplatin injected rats. This electrophysiological mechanism reflects that the peripheral pain induced by third generation platinum based agents is related to the hyperexcitability of spinal WDR neurons, and that decreasing their increased activities can lead to the attenuation of pain [21]. Downregulation of glial activation and/or cytokines [14] may play a role in this pain decreasing effect of CA. Subsequent in-depth research into its molecular mechanism is required.

## 5. Conclusions

Taken altogether, our results show that intraperitoneal administration of CA can alleviate the cold and mechanical allodynia induced by single oxaliplatin injection in rats. Moreover, this action of CA is related to the attenuation of spinal WDR neurons’ firings increased by oxaliplatin treatment. Based on these results, we suggest that CA has a potential to be an alternative to the current analgesic drug used to treat oxaliplatin-induced neuropathic pain.

## 6. Patents

The contents of this article are related to a patent application in Korea (10-2018-0065562).
